# Supplementary material for: In Vitro Activities of Ceftazidime–Avibactam and Aztreonam–Avibactam at Different Inoculum Sizes of Extended-Spectrum β-Lactam-Resistant Enterobacterales Blood Isolates
Source: Antibiotics (Basel). 2021 Dec 5;10(12):1492. doi: 10.3390/antibiotics10121492 (PMC8698305; doi:10.3390/antibiotics10121492)
Supplement: Supplementary file 1 [file antibiotics-10-01492-s001.zip › Supplement_table_s1.pdf]

Table S1. Antimicrobial susceptibilities of extended β-lactam-resistant *E. coli* (n=120) and *K. pneumoniae* (n=108) isolates to five antimicrobial agents

Species	Antimicrobial agent	Inoculum size	Cumulative % of isolates with indicated MICs (mg/L)															MIC (mg/L)[a]		S (%)[b]
			≤0.03	0.06	0.125	0.25	0.5	1	2	4	8	16	32	64	128	256	≥512	MIC_{50}	MIC_{90}	
E. coli	Ceftazidime	Standard						4.2	13.3	17.5	32.5	46.7	70.0	81.7	94.2	96.7	100	32	128	17.5
		High						0.8	3.3	8.3	14.2	24.2	34.2	44.2	55.8	74.2	100	≥512	≥512	8.3
	Aztreonam	Standard					0.8		4.2	7.5	19.2	33.3	48.3	71.7	87.5	100[c]		64	≥256	7.5
		High								0.8		4.2	10.0	15.8	35.0	100[c]		≥256	≥256	0.8
	Ceftazidime-avibactam	Standard	0.8		6.7	53.3	88.3	98.3		99.2	100							0.25	1	100
		High			0.8	25.8	70.8	89.2	90.0		93.3	97.5	99.2				100	0.5	2	93.3
	Aztreonam-avibactam	Standard	0.8	22.5	78.3	95.8	96.7	97.5	98.3	99.2		100						0.125	0.25	N/A
		High		10.8	63.3	84.2	86.7	88.3		89.2	90.0	91.7	92.5	95.0	96.7	97.5	100	0.125	8	N/A
	Meropenem	Standard	85.0	94.2	96.7	97.5	99.2			100								0.03	0.06	99.2
		High	20.8	74.2	85.0	86.7	93.3			97.5	99.2		100					0.06	0.5	93.3
K. pneumoniae	Ceftazidime	Standard								6.5	9.3	13.9	19.4	25.9	32.4	51.9	100	256	≥512	6.5
		High								0.9			2.8	3.7	5.6	9.3	100	≥512	≥512	0.9
	Aztreonam	Standard					2.8		3.7	4.6	6.5	9.3	15.7	24.1	37.0	100[c]		≥256	≥256	4.6
		High						0.9								100[c]		≥256	≥256	0.9

Ceftazidime-	Standard			0.9	9.3	37.0	69.4	89.8	97.2	98.1	99.1			100			1	4	98.1
avibactam	High				4.6	22.2	38.0	62.0	80.6	90.7	94.4	96.3	98.1		100		2	8	90.7
Aztreonam-	Standard	0.9	15.7	54.6	79.6	87.0	91.7	96.3	98.1		99.1	100					0.125	1	N/A
avibactam	High		4.6	20.4	34.3	42.6	44.4	48.1	52.8	79.6	82.4	85.2	86.1	88.0	93.5	100	4	256	N/A
Meropenem	Standard	38.0	73.1	81.5	89.8	92.6		93.5	94.4	96.3		97.2	100				0.06	0.5	92.6
	High	3.7	9.3	18.5	23.1	49.1	63.9	75.9	79.6		88.0	90.7	100				1	32	63.9

[a] 50% and 90%, MICs at which 50% and 90% of isolates are inhibited, respectively.

[b] The CLSI susceptibility breakpoint was used; ceftazidime, ≤4 mg/L; aztreonam, ≤4 mg/L; ceftazidime-avibactam, ≤8/4 mg/L; meropenem, ≤1 mg/L; no breakpoint criteria have been defined for aztreonam-avibactam.

[c] MIC is ≥ the indicated value.

MIC, minimum inhibitory concentration; N/A, not available; S, susceptible.
